# Intravascular ultrasound-based decision tree model for the optimal endovascular treatment strategy selection of femoropopliteal artery disease—results from the ONION Study-

**DOI:** 10.1186/s42155-022-00328-9

**Published:** 2022-10-06

**Authors:** Yuko Yazu, Masahiko Fujihara, Mitsuyoshi Takahara, Naoya Kurata, Aya Nakata, Hitoshi Yoshimura, Tomoaki Ito, Masashi Fukunaga, Amane Kozuki, Yusuke Tomoi

**Affiliations:** 1 Department of Medical Engineering, Kishiwada Tokushukai Hospital, Kishiwada, Japan; 2 Department of Cardiology, Kishiwada Tokushukai Hospital, Kishiwada-City Osaka, 4-27-1, Kamoricho, 596-8522 Japan; 3 Department of Medicine and Biosystemic Science, Kyushu University Graduate School of Medical Sciences, Fukuoka, Japan; 4 Department of Diabetes Care Medicine, Osaka University Graduate School of Medicine, Osaka, Japan; 5 Department of Medical Engineering, Kansai Rosai Hospital, Amagasaki, Japan; 6 Department of Medical Engineering, Morinomiya Hospital, Osaka, Japan; 7 Department of Medical Engineering, Saiseikai Nakatsu Hospital, Osaka, Japan; 8 Department of Medical Engineering, Kokura Memorial Hospital, Kitakyushu, Fukuoka Japan; 9 Cardiovascular Division, Morinomiya Hospital, Osaka, Japan; 10 Department of Cardiology, Saiseikai Nakatsu Hospital, Osaka, Japan; 11 Department of Cardiology, Kokura Memorial Hospital, Kitakyushu, Fukuoka Japan

**Keywords:** Femoropopliteal segment, Endovascular Therapy, Peripheral artery disease, Intravascular Ultrasound、Drug-Coated stent, Drug-Eluting stent, Covered stent-graft

## Abstract

**Background:**

The role of catheter-based imaging in peripheral interventions for lower extremity artery disease (LEAD) has increased with percutaneous interventions. To clarify the relation between intravascular ultrasound (IVUS) information and procedure selection strategy for endovascular treatment therapy (EVT) of the femoropopliteal artery in the real-world clinical settings wherein new endovascular technologies (NETs), including drug-coated balloon (DCB), drug-eluting stent (DES), and covered stent-graft (CS). Our retrospective multicenter analysis examined symptomatic 970 patients treated by EVT for de novo femoropopliteal lesions with IVUS guidance. The decision tree analysis was performed retrospectively to determine the association of IVUS and angiography parameters with the strategy selection of endovascular procedures. We divided the study population according to the developed tree, and identified the most popular strategy selection in each subgroup. We finally examined whether the restenosis risk would be different among respective subgroups of the tree.

**Results:**

During the study periods, plain old balloon angioplasty, DCB, and bare nitinol stent were most frequently selected (25.3%, 23.9%, and 23.8%, respectively). The drug-eluting stent (DES), covered stent (CS), and spot stent strategies were used in 7.3%, 11.5%, and 8.1%. NETs had the lowest restenosis risk in the overall population. The decision tree had a depth of six branches and divided the patients into 11 subgroups by IVUS and angiography parameters. The restenosis rate was similarly low among these 11 subgroups when the most popular NET in each subgroup was selected (*P* = 0.94).

**Conclusions:**

The use of IVUS data along with angiography data would standardize the selection of endovascular procedures and can improve patency outcomes if NETs are used properly.

## Background

Endovascular therapy (EVT) for lower extremity artery disease (LEAD) has been widely applied. Notably, atherosclerotic femoropopliteal artery disease is increasingly being treated with an endovascular approach supported by many evidence and guidelines (Aboyans et al. 2018; Allan et al. 2022; Bausback et al. 2019). Recently, new endovascular technologies (NETs), including drug-coated balloon (DCB), drug-eluting stent (DES), and covered stent (CS), have improved clinical outcomes of EVT, and the indication of EVT has been expanded to more complex lesions. The role of catheter-based imaging in EVT for LEAD has increased.　Intravascular ultrasound (IVUS)-guided intervention, a common strategy for femoropopliteal interventions, is used to evaluate the target artery, monitor guidewire position, assess plaque morphology and calcification, measure the precise vessel diameter, evaluate dissection and device selection, and determine the procedure endpoint (Bosiers et al. 2009). Since the beginning of the intervention era, additional IVUS parameters have been advocated to improve clinical outcomes^56^. However, it remained unclear whether IVUS information would be utilized for the selection of endovascular procedures. Furthermore, whether the selection would lead to optimal patency outcomes was also unclear. This study aimed to clarify the relationship between the IVUS information and the selection of endovascular procedures in the real-world clinical settings wherein NETs were clinically available. The patency outcomes according to the procedure selection was also investigated.

## Methods

### Study design and patient selection

The ONION (the Optimal eNdovascular treatment strategy for femoropopliteal artery disease controlled by Intravascular ultrasOund evaluatioN) study was a retrospective multicenter clinical investigation that analyzed patients treated for symptomatic femoropopliteal de novo occlusive lesions between January 2016 and December 2018 at five centers across Japan. The study examined 970 consecutive patients (718 male and 252 female patients; mean age: 74.1 ± 9 years) who had intermitted claudication symptoms classified as grades 2–3 based on the Rutherford scale (Fujihara et al. 2019) and successfully underwent femoropopliteal EVT with IVUS guidance. The study’s main inclusion criterion was the presence of single or sequential de novo lesions (≥ 70% diameter stenosis or occlusion) of the superficial femoral artery, proximal popliteal artery, or both with a reference vessel diameter of 3–7 mm by angiographic analysis. Significant aortoiliac lesions were treated simultaneously with or before femoropopliteal treatment. Exclusion criteria encompassed the presence of simultaneous lesions of the common femoral artery and the lack of baseline IVUS data. Cases of atherectomy device use were also excluded. Because no atherectomy device was commercially available during this study period.

The study was conducted according to the Declaration of Helsinki and was approved by the participating institutions’ institutional review boards.

### Endovascular therapy procedure and follow-up

Medications such as aspirin, clopidogrel, oral anticoagulants, and heparin were administered according to local hospital policy and physicians’ discretion. A sheath was inserted into the ipsilateral or contralateral common femoral artery. A baseline angiography was performed to evaluate the lesion location, lesion length, and stenotic or chronic total occlusion (CTO). After infusion of 5000–8000 units of heparin, a guidewire was used to cross the lesion, and IVUS evaluation was assessed for whole lesions. After baseline IVUS evaluation, an optimal-sized conventional balloon and/or scoring/cutting balloon were applied to dilate the lesion. After that, commercially available stent implantation, DCB dilatation, or bare balloon alone procedures were performed. The treatment strategies were classified into plain old balloon angioplasty (POBA), bare nitinol stent (BNS), DCB, DES, CS, and spot bare nitinol stent implantation with POBA (spot). Technical success was defined as < 30% residual stenosis without flow-limiting severe dissection after stent implantation or after the balloon angioplasty strategy (Fujihara et al. 2022). Follow-up clinical evaluations were conducted six and 12 months after the procedure. Follow-up evaluations for clinical symptoms and lesion patency were assessed using duplex ultrasound. The restenosis was defined as an at-rest peak systolic velocity of ≥ 2.5 on duplex ultrasound without reintervention (Hong et al. 2015).

### Intravascular ultrasound protocol

In all patients having an IVUS-guided procedure, the pre/post IVUS images were recorded using a 20–60 MHz transducer connected to a console system [60 MHz AltaView IVUS, Terumo, Tokyo, Japan; 40–60 MHz Opticross IVUS, Boston Scientific, Marlborough, MA, USA; and 20-MHz Eagle Eye or Eagle Eye Platinum catheter, Philips Volcano, Rancho Cordova, CA, USA)]. The interventional cardiologist advanced the IVUS catheter beyond the lesion to capture images with manual pullback. If the IVUS catheter could not cross the lesion, it was dilated using a ≤ 2.0-mm balloon to allow the catheter to pass.

IVUS imaging was used to evaluate the guidewire route, measure vessel diameters and areas, maximum circumferential distribution of calcium, and vessel dissection pattern after pre balloon angioplasty (Iida et al. 2014). The guidewire route was evaluated as all intimal space (True), partial subintimal space (Sub-True), and all subintimal space (Sub). Vessel size and area were delimited by the external elastic membrane (EEM), and the proximal–distal reference vessel and lesion defined as the most stenotic segment were identified. Reference segments were selected as the most normal-looking sections within 10 mm on either side of the lesion. The lumen diameter and area were also evaluated. The maximum calcification site was defined as the area in which calcification was circumferentially most extensively distributed (Iida et al. 2015).

The severity of vessel dissection after the initial balloon angioplasty procedure was assessed. If several dissection patterns were evident in a single lesion, the worst was used for the analysis. Six grades of dissection (A1, A2, B1, B2, C1, and C2) were employed according to the iDissection classification criteria. This classification combines the depth of injury from intima to adventitia with the circumference of dissection. The iDissection category is defined as (A1) < 180°, intima; (A2) ≥ 180°, intima; (B1) < 180°, media; (B2) ≥ 180°, media; (C1) < 180°, adventitia; (C2) ≥ 180°, adventitia (Iida et al. 2019).

### Outcomes

The primary study outcome was the influence of IVUS and angiography parameters for basic procedure strategy selection. The basic procedure strategy included plain old balloon angioplasty (POBA), dug coated balloon (DCB), bare nitinol stent (BNS), drug eluting stent (DES), the covered stent-graft (CS), and spot bare nitinol stent implantation with POBA (SPOT). This outcome was analyzed by decision tree analysis. The secondary outcomes were the six months and one-year restenosis rates associated with the basic procedure strategy. The optimal procedure strategy in each subgroup was also evaluated.

### Statistical analyses

If not otherwise mentioned, data were presented as means and standard deviations for continuous variables or percentages for discrete variables. *P* < 0.05 was considered statistically significant, and 95% confidence intervals were reported where appropriate. To explore IVUS and angiography findings associated with the selection of endovascular procedures, we first developed a decision tree. We adopted the decision tree analysis because this analysis can handle nonlinear relationships, explore potential thresholds of covariates, and flexibly treat potential interaction effects among covariates. The candidates for associated factors were lesion length, CTO, popliteal alone, subintimal guidewire passage, distal EEM diameter, distal lumen diameter, calcification, and post-angioplasty dissection. The intergroup difference in the proportion of restenosis was tested by the chi-squared test. Missing data were addressed using the multiple imputation method. All statistical analysis was performed using R version 3.6.1 (R Development Core Team).

## Results

Baseline characteristics of the study population are presented in Table 1. The mean age was 74.1 ± 8.5 years, and the prevalence of hypertension, current smoking habits, diabetes mellitus, and chronic renal failure were 77%, 27%, 54%, and 30%, respectively. The average lesion length was 144 mm, and the percentage of CTO was 43%. The Opticross IVUS, AltaView IVUS, and Volcano IVUS were used for 40.3%, 39.2%, and 20.2%, respectively.

**Table 1:** Baseline demographic, clinical, and lesion characteristics of 970 patients

Age (years old)	74.1 ± 8.5
Male (%)	718 (74.0)
Hypertension(%)	750 (77.0)
Diabetes (%)	527 (54.3)
Dyslipidemia (%)	483 (49.8)
Current Smoking (%)	263 (27.1)
Obesity (%) BMI > 25^a^	226 (23.2)
CRF (%)^a^	294 (30.3)
ESRD^a^ on dialysis	230 (23.7)
Rutherford II	291 (30.0)
Rutherford III	679 (70.0)
ABI^a^	0.65 ± 0.20
CTO^a^ (%)	369 (38.0)
Lesion length (mm)	144.4 ± 01
Popliteal artery alone (%)	56 (5.8)
Aspirin (%)	851 (87.7)
Clopidgrel (%)	747 (77.0)
Cilostazol (%)	232 (23.8)
OAC^a^ (%)	134 (13.8)
Statin (%)	397 (40.9)
Opticross IVUS (%)	391 (40.3)
AltaView IVUS (%)	380 (39.2)
Volcano IVUS (%)	196 (20.2)
Other IVUS (%)	3 (0.3)

^a^ *ABI* Ankle brachial index, *BMI* Body mass index, *CRF* Chronic renal failure, *CTO* Chronic total occlusion, *ESRD* End-stage renal dysfunction, *OAC* Oral anticoagulation therapy, *IVUS* Intravascular ultrasound

**Fig. 1:**
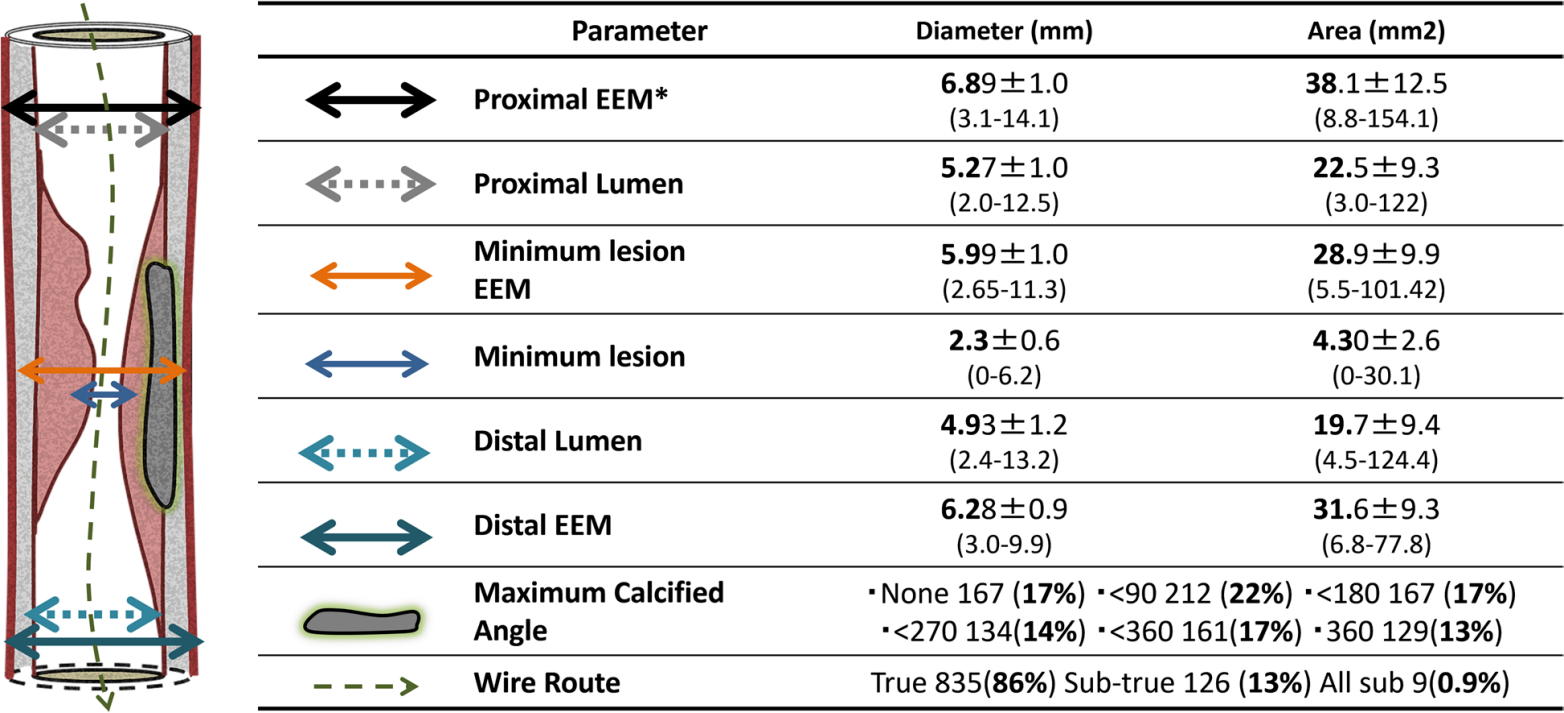
Baseline intravascular ultrasound data. EEM: external elastic membrane

The baseline IVUS parameters are shown in Fig. 1. The distal reference EEM was 6.28 ± 0.90 mm, and the distal reference lumen diameter was 4.93 ± 1.20 mm. The cases with calcification were 83%, and 13% of patients had round shape 360-degree calcification. The percentages of guidewire passage routes that were all true, sub-true, and all sub were 86%,13%, and 0.9%.

**Fig. 2:**
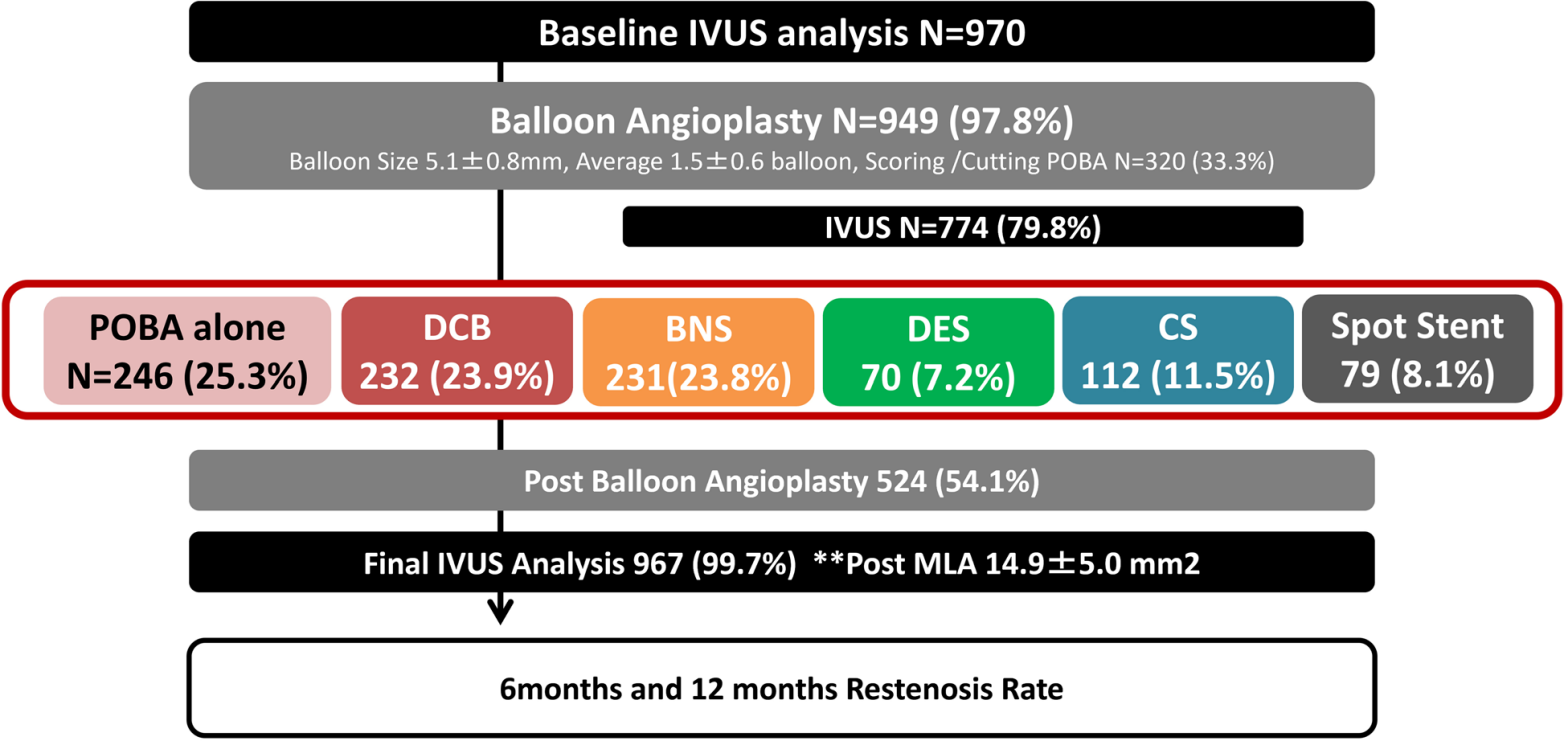
The scheme of study. Patient enrollment numbers: consented (970), plain old balloon angioplasty: POBA (246), drug-coated balloon: DCB (232), bare nitinol stent: BNS (231), drug-eluting stent: DES (70), covered stent: CS (112), and spot stent (79). IVUS: intravascular ultrasound, MLA: Minimum Lumen Area

In this study period, POBA, BNS, and DCB were most frequently selected (25.3%, 23.9%, and 23.8%, respectively), whereas DES, CS, and spot stent strategies were used in 7.3%, 11.5%, and 8.1%, respectively (Fig. 2). Table 2 shows the lesion characteristics according to the standard procedure strategy. After the pre balloon angioplasty, grade B2 and C dissection occurred in 26% and 13% of the cases as severe dissection.

**Table 2:** The lesion characteristics according to the standard procedure strategy by intravascular ultrasound evaluation

	**All *n* = 970**	**POBA *n* = 246**	**DCB *n* = 232**	**BNS *n* = 231**	**DES *n* = 70**	**CS *n* = 112**	**Spot *n* = 79**
Chronic total occlusion	**369 (38%)**	60 (24%)	47 (20%)	104 (45%)	22 (31%)	69 (62%)	67 (85%)
Popliteal alone	**56 (6%)**	30 (12%)	23 (10%)	3 (1%)	0 (0%)	0 (0%)	0 (0%)
Lesion length (mm)	**144 ± 102**	110 ± 88	111 ± 81	131 ± 86	93 ± 66	268 ± 85	259 ± 61
Distal EEM* diameter(mm)	**6.3 ± 0.9**	6.1 ± 1.0	6.2 ± 0.9	6.4 ± 0.9	6.6 ± 0.7	6.5 ± 1.0	6.1 ± 1.0
Distal lumen diameter(mm)	**4.9 ± 0.9**	4.7 ± 1.0	4.9 ± 1.0	5.0 ± 0.9	5.3 ± 0.9	5.0 ± 0.9	4.7 ± 0.9
*Calcification*–None	**167 (17%)**	48 (20%)	42 (18%)	35 (15%)	16 (23%)	12 (11%)	14 (18%)
< 90°	**212 (22%)**	43 (17%)	62 (27%)	49 (21%)	19 (27%)	23 (21%)	16 (20%)
< 180°	**167 (17%)**	40 (16%)	40 (17%)	36 (16%)	15 (21%)	20 (18%)	16 (20%)
< 270°	**134 (14%)**	36 (15%)	37 (16%)	28 (12%)	8 (11%)	18 (16%)	7 (9%)
< 360°	**161 (17%)**	44 (18%)	33 (14%)	32 (14%)	6 (9%)	29 (26%)	17 (22%)
360°	**129 (13%)**	35 (14%)	18 (8%)	51 (22%)	6 (9%)	10 (9%)	9 (11%)
Subintimal route	**135 (14%)**	9 (4%)	8 (3%)	39 (17%)	4 (6%)	42 (38%)	33 (42%)
Dissection after PTA*							
None/Grade A/B1	**468 (60%)**	155 (67%)	134 (62%)	101 (63%)	22 (56%)	18 (30%)	38 (59%)
Grade B2	**205 (26%)**	65 (28%)	65 (30%)	41 (25%)	10 (26%)	12 (20%)	12 (19%)
Grade C1/C2	**101 (13%)**	12 (5%)	18 (8%)	19 (12%)	7 (18%)	31 (51%)	14 (22%)
Missing data	**196 (20%)**	14 (6%)	15 (6%)	70 (30%)	31 (44%)	51 (46%)	15 (19%)

*BNS* Bare nitinol stent, *CS* Covered stent, *DCB* Drug-coated balloon, *DES* Drug-eluting stent, *EEM* External elastic membrane, *POBA* Plain old balloon angioplasty, *PTA* Percutaneous transluminal angioplasty

**Fig. 3:**
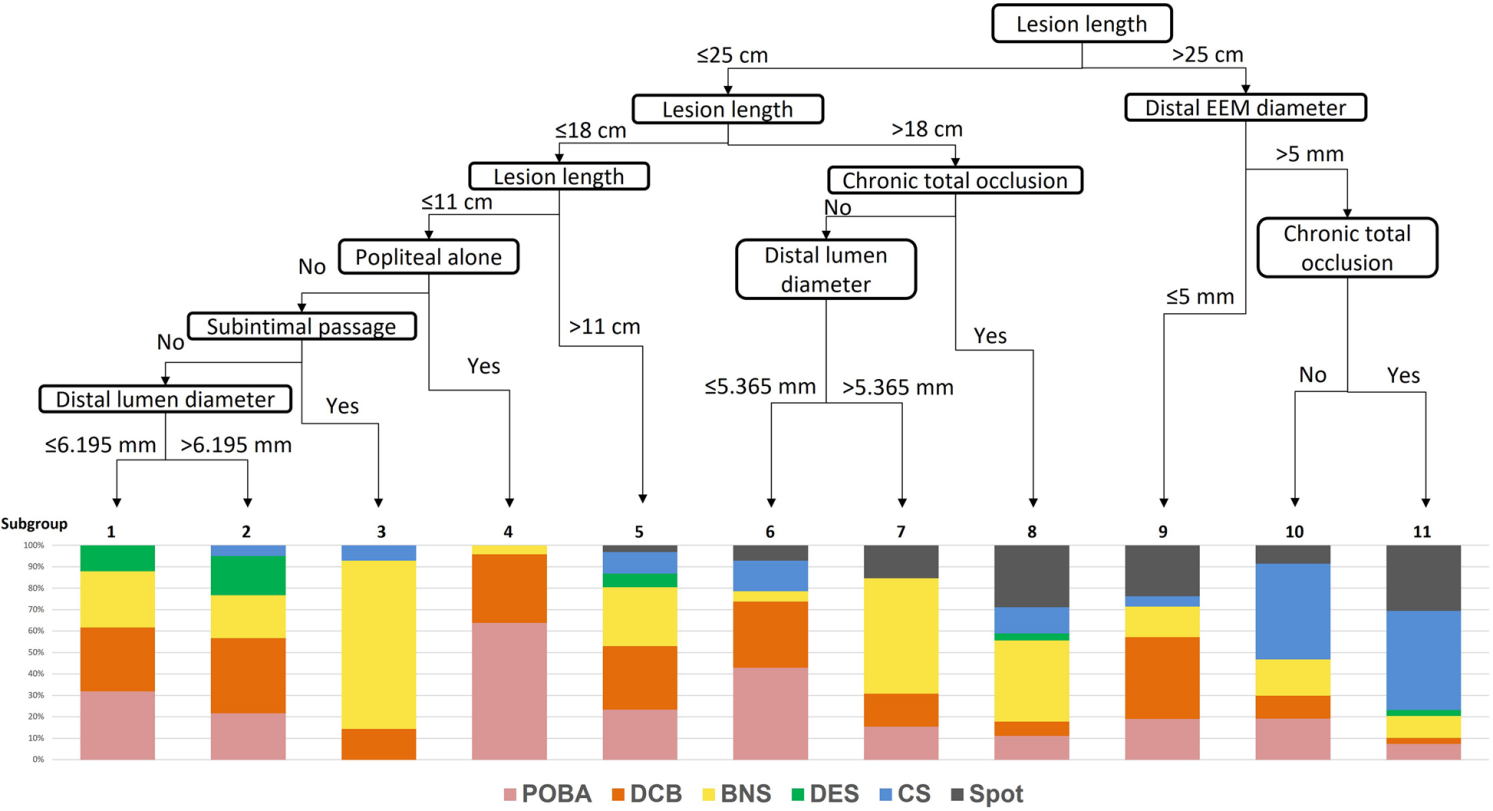
Decision tree analysis. BNS: bare nitinol stent, CS: covered stent, DCB: drug-coated balloon, DES: drug-eluting stent, EEM: external elastic membrane, POBA: plain old balloon angioplasty

Figure 3 shows the developed decision tree for the selection of endovascular procedures retrospective. The tree had a depth of six branches and divided patients into 11 subgroups. The first branch was for a lesion length > 25 cm or ≤ 25 cm. In lesions of > 25 cm, distal EEM diameter of > 5 mm was associated with a preferable use of CS, whereas the diameter of ≤ 5 mm was associated with a preferable use of DCB. Spot stent strategy was also preferably selected in long lesions with CTO or distal EEM diameter ≤ 5 mm. In contrast, shorter lesion, open vessel, and true lumen guidewire passage were associated with a preference for the balloon alone procedure (POBA or DCB). The calcification and dissection pattern after post percutaneous transluminal angioplasty (PTA) was not identified as factors associated with the procedure selection. In subgroups 1–7 and 9, DCB was the most popular NETs. In subgroups 1, 8, and 11, CS was the most popular NETs.

**Fig. 4:**
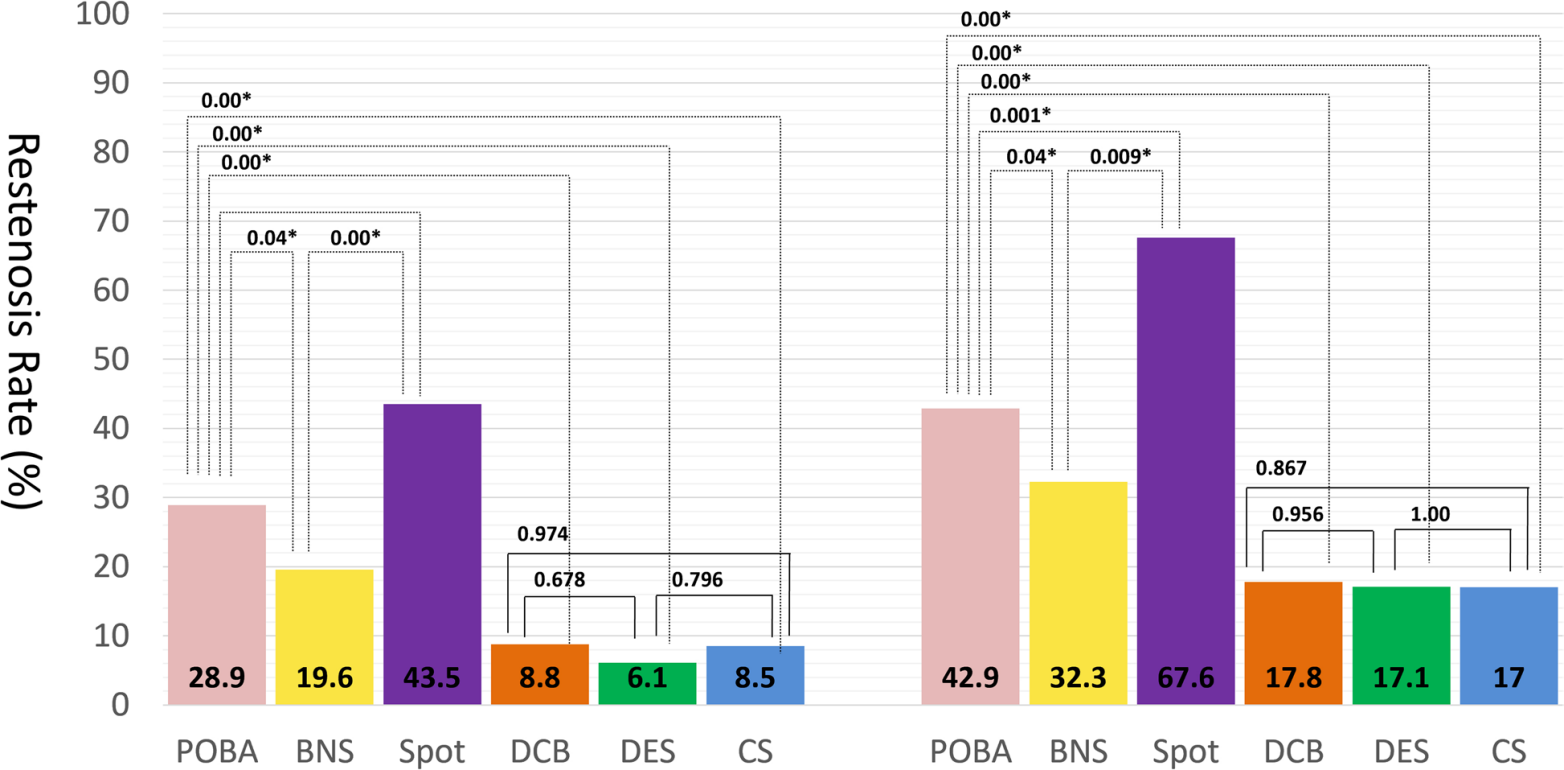
The restenosis rate of six months and one year by the strategy. The low restenosis rate was proven in DCB, DES, and CS groups. Otherwise, the restenosis rates in the POBA, BNS, and spot stent strategy groups were significantly higher. BNS: bare nitinol stent, CS: covered stent, DCB: drug-coated balloon, DES: drug-eluting stent, POBA: plain old balloon angioplasty

**Fig. 5:**
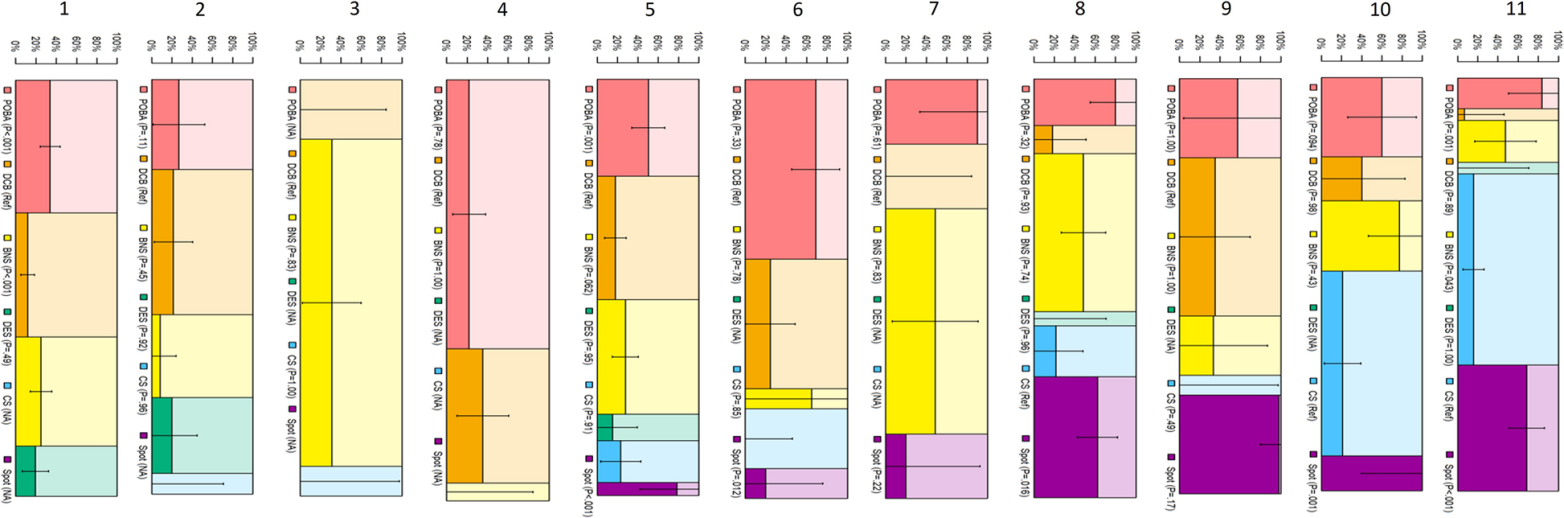
The restenosis rate applied to the 11 categories. BNS: bare nitinol stent, CS: covered stent, DCB: drug-coated balloon, DES: drug-eluting stent, POBA: plain old balloon angioplasty

**Fig. 6:**
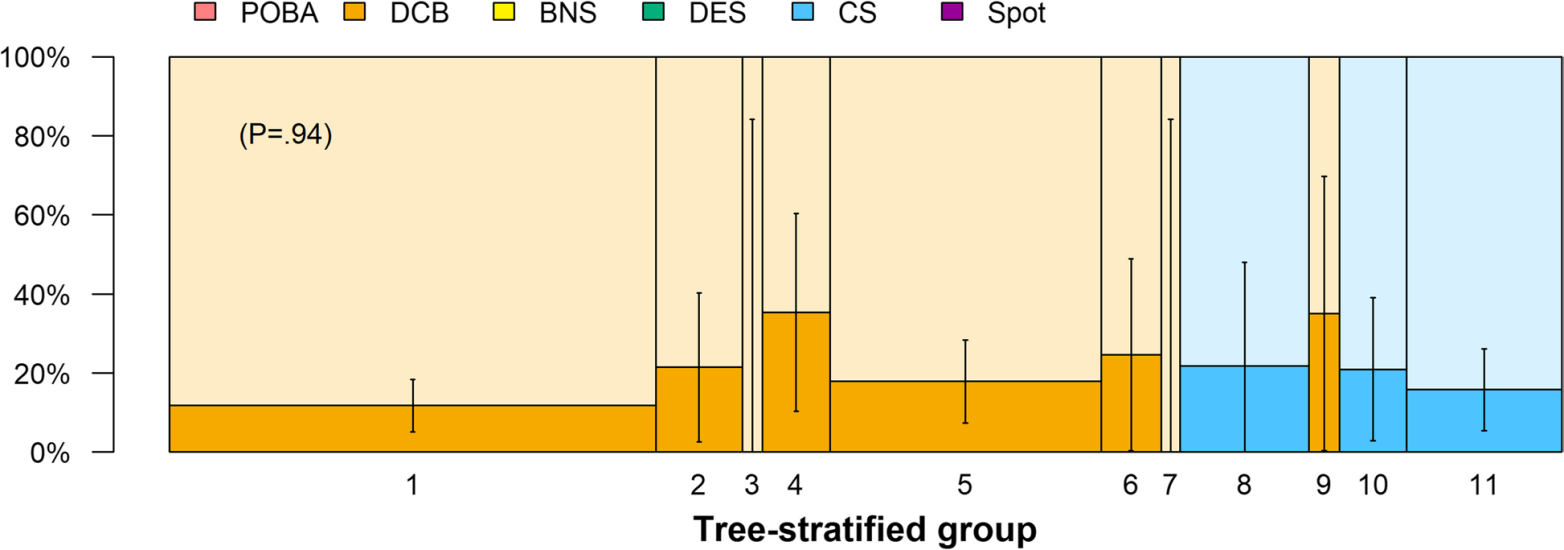
The restenosis rate of reference strategy in each category. The most popular strategy set the reference in DCB, DES, and CS. BNS: bare nitinol stent, CS: covered stent, DCB: drug-coated balloon, DES: drug-eluting stent, POBA: plain old balloon angioplasty

The restenosis rate at six months and one year by endovascular procedures in the overall population is shown in Fig. 4. NETs, i.e., DCB, DES, and CS groups had the lowest restenosis risk, whereas POBA, BNS, and spot stent strategy groups were significantly higher restenosis rate. The restenosis rates of each endovascular procedure in the 11 subgroups of the tree are shown in Fig. 5. In each subgroup, endovascular strategies other than NETs did not have a lower restenosis rate than the most popularly selected NET. As shown in Fig. 6, The restenosis rate was similarly low among these 11 subgroups when the most popular NET in each subgroup was selected (*P* = 0.94). These results indicate that favorable patency was maintained in cases in which IVUS information was used in addition to angiographic findings, and appropriate device selection was made from NETs. To begin with, PTA and BNS and their combined therapy had poor results even with IVUS.

## Discussion

The current study analyzed 970 femoropopliteal IVUS-supported EVTs and revealed the relationship between the IVUS information and the selection of endovascular procedures. This IVUS data may also be useful as basic information on femoropopliteal lesions treated in routine clinical practice.

As the expansion of indication for peripheral intervention, imaging technology is widely used. IVUS is a reliable imaging modality that reveals vast information during the procedure process. Iida et al. reported that IVUS use in femoropopliteal stenting appears to be associated not only with the procedure’s success but also with a higher primary patency rate^56^. Previous reports were based on the IVUS evaluation methodology, such as vessel size, lumen area, wire route, calcification, lesion morphology, and vessel dissection after balloon angioplasty (Iida et al. 2021; Kozuki et al. 2021; Kurata et al. 2020; Miki et al. 2020a; Miki et al. 2020b). Some parameters that influenced patency outcomes were shown, including calcification, vessel dissection, and post stent area and position (Iida et al. 2015; Mori et al. 2017; Norgren et al. 2007). Many post-hoc analyses accumulated the IVUS data by the individual finalize devices.

Except for these mentioned cases, no reliable data are available on how the operators use the IVUS information to the fullest extent. In particular, data are lacking for completing the procedure strategy selection when DCB, DES, CS, and BNS are considered.

In this study period, the basic procedure strategy was drastically changed due to the approval of a NETs in this territory. POBA alone, DCB, BNS, DES, CS, and spot stent strategy as BNS plus POBA were performed in this period. The first half, POBA alone and BNS were mainly used. The latter half, NETs were mainly used. The first polymer-free paclitaxel-coated stent, Zilver PTX stent (Cook Medical, Bloomington, IN, USA), was approved in 2012. The spot stent strategy (BNS with POBA) gained popularity between 2015 and 2017 (Rutherford and Becker 1991). However, the strategy was later found to be not so effective (Shammas et al. 2018a) and became unpopular at the end of 2016, when VIABAHN CS (W. L. Gore & Associates, Flagstaff, Ariz) and DCB (IN. PACT Admiral, Medtronic, Santa Rosa, CA, USA and LUTONIX, BD, Peripheral Interventions, Tempe, Arizona) obtained clinical approval. These approvals had a significant influence on the selection of the treatment strategy.

Our decision tree analysis identified how the operator’s selection procedure strategy is associated with the IVUS information. The data were finally divided into 11 subgroups, and the first split was associated with lesion length. In long lesion cases > 25 cm, the influenced parameters were the vessel size and total occlusion or vessel size. For medium-size vessel and occlusion cases, the CS strategy was performed. This strategy selection for long CTO lesions was also supported by the clinical evidence from VIABAHN studies (Shammas et al. 2018b).

On the contrary, short lesion cases (≤ 11 cm) led to the balloon alone procedure. In these lesion populations, DCB had shown excellent patency outcomes from extensive clinical evidence (Shammas et al. 2019). Otherwise, in the subintimal guidewire passage situation, the most popular strategy is BNS; whether either the true lumen or subintimal angioplasty is a better solution is still debatable.

In the medium length (> 18 cm, ≤ 25 cm) cases, BNS and DCB were mainly used. It is consistent with the randomized controlled trial of DCB and DES; there was no significant difference in this category (Tomoi et al. 2019).

Interestingly, the degree of calcification (Iida et al. 2015) and vessel dissection pattern after PTA by IVUS (Iida et al. 2019) have not influenced the operator’s decision for two reasons. First, these parameters from IVUS were also predictive only of angiography images. Second, a common understanding of IVUS evaluation was not established yet. Therefore, these results do not preclude the need for IVUS evaluation of calcification and vessel dissection.

After the procedure strategy selection in NETs, the restenosis rate was acceptably low at six months and 12 months. Otherwise, PTA alone, BMS, and the spot stent strategy had a significantly high restenosis rate. The outcomes could be adequately explained by the transition to an improved device. In the later period of this study, NETs were mainly used. For the restenosis rate at 12 months, the decision tree analysis revealed that there was no significant difference in the use of the most selected approach for each subgroup. The results suggest that EVT with IVUS is highly useful in cases with NETs. Conversely, EVT with POBA, BNS, and both Spot stents may be less objectionable due to poor patency to begin with. In a latest RCT, Allan RB et al. demonstrated that the use of IVUS reduces restenosis (Fujihara et al. 2017). In this study, significance was also demonstrated, mainly in the DCB group, which is consistent with the present study.

These results are only informative for this period of treatment devices and methods. Future improvements in new devices and treatment methods may affect the results. Nevertheless, this study period was dominated by the use of NETs, which is likely to become the first choice in practice in the near future. We believe that this will be a useful reference for some time to come.

Although many IVUS data have been published in recent years, it remains to be discussed whether the routine use of IVUS is economically and procedurally justified. More appropriate and conditionally appropriate use of IVUS is expected. Further research in this regard is warranted.

### Limitation

There are some limitations to this study. First, This study was a single-arm study without a control arm. Also, this study represents a nonrandomized investigation with selection bias. The strategies for EVT were based on the discretion of the treating physician. These factors may have influenced the clinical outcome and results. The study lacks any core laboratory-based analysis, which may also be considered a limitation.

## Conclusion

The use of IVUS data standardizes the procedure selection and can improve patency outcomes for femoropopliteal artery disease. The patency outcomes of NETs strategies were favorable and not significantly different based on the decision tree analysis model.

## Data Availability

The datasets used and/or analyzed during the current study are available from the corresponding author on reasonable request.

## Abbreviations

IVUS: Intravascular ultrasound
EVT: Endovascular treatment therapy
NETs: New endovascular technologies
DCB: Drug-coated balloon
DES: Drug-eluting stent
CS: Covered stent-graft
CTO: Chronic total occlusion
POBA: Balloon angioplasty
BNS: Bare nitinol stent
EEM: External elastic membrane

## References

[CR1] Aboyans V, Ricco JB, Bartelink M, Björck M, Brodmann M, Cohnert T, Collet JP, Czerny M, De Carlo M, Debus S, Espinola-Klein C, Kahan T, Kownator S, Mazzolai L, Naylor AR, Roffi M, Röther J, Sprynger M, Tendera M, Tepe G, ESC Scientific Document Group (2018). ESC guidelines on the diagnosis and treatment of peripheral arterial diseases, in collaboration with the European Society for Vascular Surgery (ESVS): document covering atherosclerotic disease of extracranial carotid and vertebral, mesenteric, renal, upper and lower extremity arteries endorsed by: the European Stroke Organization (ESO)the task force for the diagnosis and treatment of peripheral arterial diseases of the European Society of Cardiology (ESC) and of the European Society for Vascular Surgery (ESVS). Eur Heart J.

[CR2] Allan RB, Puckridge PJ, Spark JI, Delaney CL (2022). The impact of intravascular ultrasound on femoropopliteal artery endovascular interventions: a randomized controlled trial. JACC Cardiovasc Interv.

[CR3] Bausback Y, Wittig T, Schmidt A, Zeller T, Bosiers M, Peeters P, Brucks S, Lottes AE, Scheinert D, Steiner S (2019). Drug-eluting stent versus drug-coated balloon revascularization in patients with femoropopliteal arterial disease. J Am Coll Cardiol.

[CR4] Bosiers M, Torsello G, Gissler HM, Ruef J, Müller-Hülsbeck S, Jahnke T, Peeters P, Daenens K, Lammer J, Schroë H, Mathias K, Koppensteiner R, Vermassen F, Scheinert D (2009). Nitinol stent implantation in long superficial femoral artery lesions: 12-month results of the DURABILITY I study. J Endovasc Ther.

[CR5] Fujihara M, Takahara M, Sasaki S, Nanto K, Utsunomiya M, Iida O, Yokoi Y (2017). Angiographic dissection patterns and patency outcomes after balloon angioplasty for superficial femoral artery disease. J Endovasc Ther.

[CR6] Fujihara M, Kozuki A, Tsubakimoto Y, Takahara M, Shintani Y, Fukunaga M, Iwasaki Y, Nakama T, Yokoi Y (2019). Lumen gain after endovascular therapy in calcified superficial femoral artery occlusive disease assessed by intravascular ultrasound (CODE Study). J Endovasc Ther.

[CR7] Fujihara M, Kurata N, Yazu Y, Mori S, Tomoi Y, Horie K, Nakama T, Tsujimura T, Nakata A, Iida O, Sonoda S, Torii S, Ishihara T, Azuma N, Urasawa K, Ohki T, Komori K, Kichikawa K, Yokoi H, Nakamura M. (2022). Clinical expert consensus document on standards for lower extremity artery disease of imaging modality from the Japan Endovascular Treatment Conference. Cardiovasc Interv Ther. Jul 19. 10.1007/s12928-022-00875-x. Epub ahead of print. Erratum in: Cardiovasc Interv Ther. 2022 Aug 11;: PMID: 35852760. 10.1007/s12928-022-00875-x 35852760

[CR8] Hong SJ, Ko YG, Shin DH, Kim JS, Kim BK, Choi D, Hong MK, Jang Y (2015). Outcomes of spot stenting versus long stenting after intentional subintimal approach for long chronic total occlusions of the femoropopliteal artery. JACC Cardiovasc Interv.

[CR9] Iida O, Takahara M, Soga Y, Suzuki K, Hirano K, Kawasaki D, Shintani Y, Suematsu N, Yamaoka T, Nanto S, Uematsu M (2014). Efficacy of intravascular ultrasound in femoropopliteal stenting for peripheral artery disease with TASC II class A to C lesions. J Endovasc Ther.

[CR10] Iida O, Takahara M, Soga Y, Nakano M, Yamauchi Y, Zen K, Kawasaki D, Nanto S, Yokoi H, Uematsu M, ZEPHYR Investigators (2015). 1-year results of the ZEPHYR registry (Zilver PTX for the femoral artery and proximal popliteal artery): predictors of restenosis. JACC Cardiovasc Interv.

[CR11] Iida O, Soga Y, Urasawa K, Saito S, Jaff MR, Wang H, Ookubo H, Yokoi H, MDT-2113 SFA Japan Investigators (2019). Drug-coated balloon versus uncoated percutaneous transluminal angioplasty for the treatment of atherosclerotic lesions in the superficial femoral and proximal popliteal artery: 2-year results of the MDT-2113 SFA Japan randomized trial. Catheter Cardiovasc Interv.

[CR12] Iida O, Takahara M, Soga Y, Yamaoka T, Nanto S, Kuratani T, Sakata Y, Mano T (2021). One-year outcomes of heparin-bonded stent-graft therapy for real-world femoropopliteal lesions and the association of patency with the prothrombotic state based on the prospective, observational, multicenter viabahn stent-graft placement for femoropopliteal diseases requiring endovascular therapy (VANQUISH) Study. J Endovasc Ther.

[CR13] Kozuki A, Takahara M, Shimizu M, Kijima Y, Nagoshi R, Fujiwara R, Shibata H, Suzuki A, Soga F, Miyata T, Sakamoto Y, Seo H, Asada H, Isawa K, Higuchi K, Shite J (2021). Outcomes of dissection angles as predictor of restenosis after drug-coated balloon treatment. J Atheroscler Thromb.

[CR14] Kurata N, Iida O, Asai M, Masuda M, Okamoto S, Ishihara T, Nanto K, Mano T (2020). Factors influencing in-stent occlusion after femoropopliteal artery stent placement with intravascular ultrasound evaluation. J Vasc Interv Radiol.

[CR15] Miki K, Tanaka T, Yanaka K, Yoshihara N, Kimura T, Imanaka T, Akahori H, Ishihara M (2020). Influence of self-expanding paclitaxel-eluting stent sizing on neointimal hyperplasia in superficial femoral artery lesions. Circ J.

[CR16] Miki K, Fujii K, Tanaka T, Yanaka K, Yoshihara N, Nishimura M, Sumiyoshi A, Horimatsu T, Imanaka T, Fukunaga M, Akahori H, Masuyama T, Ishihara M (2020). Impact of IVUS-Derived vessel size on midterm outcomes after stent implantation in femoropopliteal lesions. J Endovasc Ther.

[CR17] Mori S, Hirano K, Ito Y, Yamawaki M, Araki M, Kobayashi N, Takimura H, Sakamoto Y, Tsutsumi M, Takama T, Honda Y, Tokuda T, Makino K, Shirai S (2017). Clinical outcomes of the intraluminal approach for long occlusive femoropopliteal lesions assessed by intravascular ultrasound. J Atheroscler Thromb.

[CR18] Norgren L, Hiatt WR, Dormandy JA, Nehler MR, Harris KA, Fowkes FG, TASC II Working Group (2007). Inter-society consensus for the management of peripheral arterial disease (TASC II). J Vasc Surg.

[CR19] Rutherford RB, Becker GJ (1991). Standards for evaluating and reporting the results of surgical and percutaneous therapy for peripheral arterial disease. J Vasc Interv Radiol.

[CR20] Shammas NW, Torey JT, Shammas WJ (2018). Dissections in peripheral vascular interventions: a proposed classification using intravascular ultrasound. J Invasive Cardiol.

[CR21] Shammas NW, Torey JT, Shammas WJ, Jones-Miller S, Shammas GA (2018). Intravascular ultrasound assessment and correlation with angiographic findings demonstrating femoropopliteal arterial dissections post atherectomy: results from the idissection study. J Invasive Cardiol.

[CR22] Shammas NW, Radaideh Q, Shammas WJ, Daher GE, Rachwan RJ, Radaideh Y (2019). The role of precise imaging with intravascular ultrasound in coronary and peripheral interventions. Vasc Health Risk Manag.

[CR23] Tomoi Y, Soga Y, Takahara M, Fujihara M, Iida O, Kawasaki D, Ando K (2019). Spot stenting versus full coverage stenting after endovascular therapy for femoropopliteal artery lesions. J Vasc Surg.

